# Claudin 18.2 Expression in Cholangiocarcinoma: Clinicopathological Heterogeneity, Prognostic Significance, and Impact of Positivity Thresholds

**DOI:** 10.3390/jcm15176621

**Published:** 2026-08-27

**Authors:** Tugba Toyran, Kivilcim Eren Ates, Ferhat Can Piskin, Tolga Köşeci, Hatice Asoglu, Yusuf Kemal Arslan, Ugur Topal, Arbil Acikalin

**Affiliations:** 1 Department of Pathology, Faculty of Medicine, Cukurova University, Adana 01330, Turkey; kivilcimerenates@hotmail.com (K.E.A.); arbilacikalin@gmail.com (A.A.); 2 Department of Radiology, Faculty of Medicine, Cukurova University, Adana 01330, Turkey; ferhathoca86@gmail.com; 3 Department of Medical Oncology, Faculty of Medicine, Cukurova University, Adana 01330, Turkey; drtolgakoseci@gmail.com (T.K.); drhatice10@gmail.com (H.A.); 4 Department of Biostatistics, Faculty of Medicine, Cukurova University, Adana 01330, Turkey; ykarslan@gmail.com; 5 Department of Surgical Oncology, Faculty of Medicine, Cukurova University, Adana 01330, Turkey

**Keywords:** cholangiocarcinoma, Claudin 18.2, prognostic biomarker

## Abstract

**Background/Objectives:** Claudin 18.2 (CLDN18.2) has emerged as a clinically actionable therapeutic target in gastrointestinal malignancies; however, its expression profile and prognostic significance in cholangiocarcinoma (CCA) remain incompletely characterized. This study investigated CLDN18.2 expression in CCA and evaluated its associations with clinicopathological characteristics and overall survival (OS). **Methods:** This retrospective study included 124 patients with histologically confirmed CCA who were diagnosed between 2010 and 2025. CLDN18.2 expression was assessed by immunohistochemistry using two predefined positivity thresholds (≥10% and ≥75% of tumor cells showing moderate-to-strong membranous staining). Associations with clinicopathological variables were analyzed, and prognostic significance was evaluated using Kaplan–Meier survival analysis and Firth’s penalized Cox regression. **Results:** Using a 10% cutoff, CLDN18.2 positivity was detected in 10.5% (13/124) of cases; using a 75% cutoff, it was detected in 4.8% (6/124) of cases. At the 75% threshold, CLDN18.2 expression was significantly associated with anatomical location (*p* = 0.013) and histological subtype (*p* = 0.002), with the highest positivity observed in perihilar CCA (30.0%) and large-duct intrahepatic CCA (21.7%). The median OS was 9.5 months. CLDN18.2 positivity was not significantly associated with mortality in a univariable Firth’s penalized Cox regression at either the 10% cutoff (hazard ratio (HR) = 1.641, 95% confidence interval (CI): 0.883–2.816; *p* = 0.112) or the 75% cutoff (HR = 1.884, 95% CI: 0.762–3.877; *p* = 0.155). In multivariable Firth’s penalized Cox regression, CLDN18.2 positivity at the 10% cutoff was independently associated with an approximately two-fold increased risk of mortality (HR = 2.072, 95% CI: 1.090–3.666; *p* = 0.028), whereas no independent prognostic significance was observed at the 75% cutoff (HR = 1.906, 95% CI: 0.768–3.951; *p* = 0.150). The absence of adjuvant therapy was independently associated with increased mortality in both models (10% cutoff: HR = 4.870, 95% CI: 1.671–13.784; *p* = 0.004; 75% cutoff: HR = 4.296, 95% CI: 1.521–11.899; *p* = 0.006). **Conclusions:** CLDN18.2 expression in CCA varies according to anatomical location, histological subtype, and positivity threshold. The relatively high expression observed in perihilar CCA, and large-duct intrahepatic CCA, and the independent prognostic significance identified at the 10% cutoff support the potential role of CLDN18.2 as a prognostic biomarker and therapeutic target in selected patients with CCA. Given the limited sample sizes in some anatomical and histological subgroups, these findings should be interpreted cautiously and validated in larger prospective multicenter studies.

## 1. Introduction

Cholangiocarcinoma (CCA) is a rare but highly aggressive malignancy arising from the biliary epithelium and is anatomically classified into three subtypes: intrahepatic (iCCA), perihilar (pCCA), and distal (dCCA) [1,2]. Over recent decades, the incidence of iCCA has steadily increased worldwide. Most patients present with advanced or unresectable disease at diagnosis. Although surgical resection remains the only potentially curative treatment, only a minority of patients are eligible for surgery, and postoperative recurrence is common, substantially limiting long-term overall survival (OS) [2,3]. The poor prognosis of CCA reflects its marked biological heterogeneity, its insidious clinical presentation, and its limited response to currently available systemic therapies.

Based on the results of the pivotal ABC-02 randomized phase III trial, gemcitabine plus cisplatin (GemCis) has long been established as the standard first-line systemic therapy for patients with advanced CCA, with limited therapeutic advances achieved for more than a decade [4]. More recently, the phase III TOPAZ-1 trial demonstrated that the addition of durvalumab to GemCis significantly improved OS compared with GemCis alone, establishing chemoimmunotherapy as the current standard first-line treatment for advanced biliary tract cancer [5]. Nevertheless, currently available molecularly targeted therapies benefit only a relatively small subset of patients. Consequently, the identification of novel therapeutic targets with the potential to benefit broader patient populations has become a major research priority.

Claudins are integral membrane proteins that constitute the backbone of epithelial tight junctions and play essential roles in maintaining epithelial polarity and regulating paracellular permeability. Claudin 18.2 (CLDN18.2), a gastric-specific isoform, is predominantly expressed under physiological conditions in differentiated epithelial cells of the gastric mucosa, where it is confined to the apical tight junction complex and is therefore inaccessible to circulating antibodies. During malignant transformation, disruption of tight junction integrity exposes CLDN18.2 across the tumor cell membrane, rendering it accessible to therapeutic monoclonal antibodies [6]. This highly restricted expression pattern in normal tissues, together with its increased surface accessibility in malignant cells, makes CLDN18.2 an attractive therapeutic target with minimal expected off-target toxicity.

On the basis of these biological characteristics, the anti-CLDN18.2 monoclonal antibody zolbetuximab induces antibody-dependent cellular cytotoxicity and complement-dependent cytotoxicity, leading to selective elimination of CLDN18.2-expressing tumor cells [6]. The phase III SPOTLIGHT [7] and GLOW [8] trials demonstrated that the addition of zolbetuximab to standard chemotherapy significantly improved progression-free survival (PFS) and OS in patients with CLDN18.2-positive, HER2-negative gastric or gastroesophageal junction adenocarcinoma, thereby establishing CLDN18.2-targeted therapy as an effective treatment strategy in gastrointestinal oncology.

Despite these advances, the clinicopathological significance and prognostic value of CLDN18.2 expression in CCA remain incompletely defined. In this retrospective cohort of 124 patients with CCA, we investigated CLDN18.2 expression at the 10% and 75% positivity thresholds, evaluated its association with anatomical location and histological subtype, and assessed its independent prognostic impact on OS.

## 2. Materials and Methods

### 2.1. Study Design and Case Selection

This retrospective cohort study included 124 consecutive patients with histologically confirmed CCA who were diagnosed in the Department of Pathology, at Cukurova University Faculty of Medicine, between 2010 and 2025. Eligible cases were identified through a review of the pathology archives and the hospital information management system. Cases with insufficient tumor tissue for immunohistochemical (IHC) analysis, inadequate tissue fixation, technical artifacts, or incomplete clinicopathological data were excluded. Demographic, clinical, and pathological data, including age, tumor size, lymph node status, lymphovascular invasion (LVI), perineural invasion (PNI), distant metastasis, and histological subtype, were retrieved from pathology reports and electronic medical records. Histological classification and tumor staging were performed according to the 5th edition of the WHO Classification of Digestive System Tumours and the 8th edition of the American Joint Committee on Cancer (AJCC) TNM staging system, respectively [1]. Pathological TNM classification was used when adequate surgical resection specimens were available, and clinical TNM classification was assigned based on available clinical and radiological findings in patients who did not undergo surgical resection.

### 2.2. Histopathological Evaluation

All hematoxylin and eosin (H&E)-stained slides were reviewed, and representative formalin-fixed, paraffin-embedded (FFPE) tissue blocks containing an adequate amount of invasive tumor were selected for IHC analysis.

### 2.3. Immunohistochemistry

IHC staining for CLDN18.2 was performed on 4 µm-thick formalin-fixed, FFPE tissue sections. Tissue sections were processed using an automated Ventana BenchMark ULTRA system (Roche Diagnostics, Tucson, AZ, USA). Deparaffinization was performed using the Ultraview DAB protocol, with the slides heated to 72 °C. Heat-induced antigen retrieval was performed using ULTRA CC1 after cell conditioning with ULTRA Conditioner at 95 °C for 8 min. A mouse monoclonal anti-CLDN18.2 antibody (clone 43-14A; Abcam, Cambridge, UK; catalog no. ab314690) was used as the primary antibody at a dilution of 1:5000 and was incubated for 1 h. Immunodetection was performed using the Ventana Ultraview Universal DAB detection system (Roche Diagnostics, Tucson, AZ, USA). For counterstaining, hematoxylin was applied and incubated for 12 min, followed by the bluing reagent for 4 min. Normal gastric mucosa, which demonstrates physiological CLDN18.2 expression, was included as a positive control in each staining run to monitor staining quality and technical consistency. The staining procedure was performed according to a standardized, automated immunohistochemistry protocol using the BenchMark ULTRA platform.

### 2.4. Evaluation of IHC Staining

**Figure 1:**
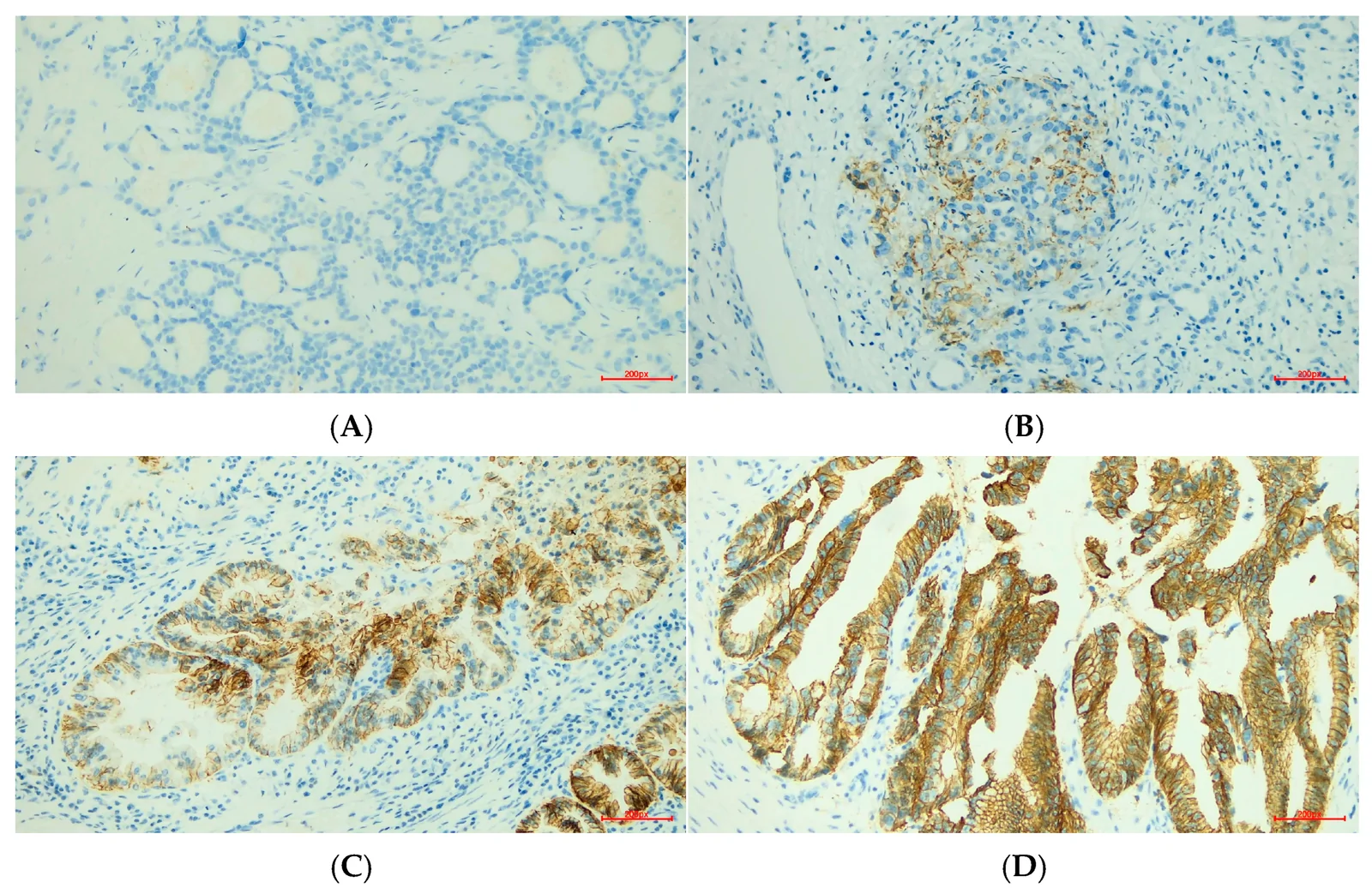
Representative examples of Claudin 18.2 (CLDN18.2) immunohistochemical (IHC) staining in cholangiocarcinoma. (**A**) Negative staining (score 0), showing no membranous staining in tumor cells. (**B**) Weak staining (score 1+), showing faint and incomplete membranous staining. (**C**) Moderate staining (score 2+), showing distinct continuous basolateral or circumferential membranous staining. (**D**) Strong staining (score 3+), showing intense continuous circumferential membranous staining in the majority of tumor cells. (IHC × 200).

CLDN18.2 expression was evaluated by light microscopy, based exclusively on membranous staining of tumor cells. The entire tumor-containing area of the selected FFPE tissue block was evaluated, rather than selecting representative hotspots, to account for potential intratumoral heterogeneity. Staining intensity was assessed using a four-tiered scale: 0 (negative), 1+ (weak), 2+ (moderate), and 3+ (strong). Weak (1+) staining was defined as faint and incomplete membranous staining; moderate (2+) staining as distinct continuous basolateral or circumferential membranous staining; and strong (3+) staining as intense, continuous circumferential membranous staining, each of which were observed in the majority of tumor cells (Figure 1). Only moderate-to-strong membranous staining (2+/3+) was considered specific for CLDN18.2 positivity.

For each case, the percentage of tumor cells exhibiting 2+/3+ membranous staining was visually estimated across the entire evaluable tumor area and recorded as a continuous percentage. Non-neoplastic stromal cells, inflammatory cells, necrotic areas, and other non-tumoral components were excluded from the assessment. CLDN18.2 staining was independently evaluated by two experienced gastrointestinal pathologists. In cases of interobserver disagreement, the final assessment was reached by consensus among the observers using a multi-headed microscope. Two predefined thresholds were subsequently applied: ≥10% of tumor cells with moderate-to-strong (2+/3+) membranous staining was considered positive for overall CLDN18.2 expression, whereas ≥75% was used to define high CLDN18.2 expression, in accordance with previously published recommendations [9,10]. The same scoring criteria were applied consistently across all cases.

### 2.5. Statistical Analysis

Categorical variables are presented as frequencies and percentages, whereas continuous variables are presented as the mean ± standard deviation or median (range), as appropriate. Comparisons between categorical variables were performed using the chi-square test or Fisher’s exact test when expected cell counts were insufficient. The normality of continuous variables was assessed using the Shapiro–Wilk test. Normally distributed variables were compared using Student’s *t*-test, whereas non-normally distributed variables were analyzed using the Mann–Whitney U test. Associations between CLDN18.2 expression and clinicopathological characteristics were evaluated separately using the 10% and 75% positivity thresholds. OS was estimated using the Kaplan–Meier method, and differences between survival curves were assessed using the log-rank test. To evaluate the prognostic significance of CLDN18.2 expression and other clinicopathological variables, univariate and multivariable Firth’s penalized Cox proportional hazards regression analyses were performed. Because complete separation occurred (no survivors were observed in the CLDN18.2-positive group), Firth’s penalized Cox proportional hazards regression was used instead of standard Cox regression to obtain more reliable estimates. Separate models were constructed using the 10% and 75% CLDN18.2 positivity thresholds. Hazard ratios (HRs) with corresponding 95% confidence intervals (95% CIs) were calculated and reported. Statistical analyses were performed using IBM SPSS Statistics version 20 (IBM Corp., Armonk, NY, USA). A two-sided *p*-value < 0.05 was considered statistically significant.

### 2.6. Ethical Considerations

The study protocol was approved by the Cukurova University Faculty of Medicine Non-Interventional Clinical Research Ethics Committee (Decision No. 157, 18 July 2025). The requirement for informed consent was waived because of the retrospective study design and the use of anonymized data. The study was conducted in accordance with the principles of the Declaration of Helsinki.

## 3. Results

### 3.1. Patient Characteristics

A total of 124 patients were included in the study, comprising 74 males (59.7%) and 50 females (40.3%). The mean age at diagnosis was 61.0 ± 10.7 years (range, 21–81 years). Based on anatomical location, 109 tumors (87.9%), 10 tumors (8.1%), and 5 tumors (4.0%) were classified as iCCA, pCCA, and dCCA, respectively. Histologically, 92 tumors (74.2%) were classified as small-duct iCCA, 23 (18.5%) as large-duct iCCA, and 9 (7.3%) as combined hepatocellular-cholangiocarcinoma (cHCC–CCA).

Multifocal tumors were identified in 84 patients (67.7%), whereas 40 patients (32.3%) had unifocal disease. The mean tumor size was 74.7 ± 39.7 mm, with a median of 80.0 mm. Detailed clinicopathological characteristics are summarized in Table 1.

CLDN18.2 expression was detected in 6 cases (4.8%) when applying the predefined 75% positivity threshold. Lowering the positivity threshold from 75% to 10% increased the CLDN18.2 positivity rate from 4.8% to 10.5%.

Among the 53 patients with distant metastases, the lung was the most frequent metastatic site (27 cases, 50.9%), followed by bone (23 cases, 43.4%) and distant lymph nodes (18 cases, 34.0%). Less frequent metastatic sites included the peritoneum (7, 13.2%), the liver (6, 11.3%), the adrenal glands (4, 7.5%), and the pleura, brain, and kidney (1 case each, 1.9%).

The mean OS was 17.4 ± 24.9 months, whereas the median OS was 9.5 months (range, 0.0–133.3 months).

### 3.2. Association Between CLDN18.2 Expression (75% Cutoff) and Clinicopathological Characteristics

The associations between CLDN18.2 expression (75% positivity threshold) and clinicopathological characteristics are summarized in Table 2. CLDN18.2 positivity was observed in 6 of 124 cases (4.8%). No significant associations were identified with sex (*p* = 0.400), age at diagnosis (*p* = 0.610), or tumor size (*p* = 0.208). A significant association was observed between CLDN18.2 expression and anatomical tumor location (*p* = 0.013). CLDN18.2 positivity was observed in 3 of 10 pCCA cases (30.0%) and in 3 of 109 iCCA cases (2.8%), whereas no positive cases were identified among patients with dCCA. Histological subtype was also significantly associated with CLDN18.2 expression (*p* = 0.002). Positivity was most frequent in large-duct iCCA (5/23, 21.7%), compared with small-duct iCCA (1/92, 1.1%); no positive cases were identified among combined cHCC–CCA cases. No significant associations were observed between CLDN18.2 expression and any of the following: histological grade (*p* = 0.730), LVI (*p* = 0.088), PNI (*p* = 0.308), multifocality (*p* = 0.085), AJCC stage (*p* = 0.378), T stage (*p* = 0.686), N stage (*p* = 0.702), M stage (*p* = 0.713), number of metastatic sites (*p* = 0.597), or portal vein invasion (*p* = 0.588).

During follow-up, 110 of the 124 patients (88.7%) died. The associations between clinicopathological characteristics and survival status are summarized in Table 3. No significant differences were observed between survivors and non-survivors with respect to sex (*p* = 0.837), age at diagnosis (*p* = 0.123), tumor size (*p* = 0.189), tumor location (*p* = 0.638), histological subtype (*p* = 0.192), histological grade (*p* = 0.214), LVI (*p* = 0.110), PNI (*p* = 0.135), or multifocality (*p* = 0.377).

In contrast, advanced tumor stage was significantly associated with mortality. According to the AJCC (8th edition) staging system, mortality increased with advancing stage, reaching 98.1% in patients with stage IV disease (*p* < 0.001). Likewise, higher T stage (*p* = 0.017), higher N stage (*p* = 0.004), and the presence of distant metastasis (M1) (*p* = 0.004) were significantly associated with poorer survival.

Surgical resection was also associated with a lower mortality rate. Mortality was 65.2% among patients who underwent resection, compared with 94.1% among those who did not (*p* < 0.001). Similarly, patients who received adjuvant therapy had a significantly lower mortality rate than those who did not (57.9% vs. 94.3%; *p* < 0.001). No significant associations were observed between survival status and portal vein invasion (*p* = 0.073), neoadjuvant therapy (*p* = 0.665), locoregional therapy (*p* = 0.936), or CLDN18.2 expression whether using the 75% (*p* = 0.370) or the 10% (*p* = 0.358) positivity threshold. At the 75% positivity threshold, all 6 patients with CLDN18.2-positive tumors had died by the time of analysis; similarly, all 13 patients classified as CLDN18.2-positive at the 10% threshold had died (Table 3). However, the limited number of CLDN18.2-positive cases within anatomical and histological subgroups, and the absence of surviving patients in these groups, precluded reliable subgroup-specific survival analyses.

Factors associated with OS were evaluated using Kaplan–Meier survival analysis and compared using the log-rank test. OS decreased significantly with advancing AJCC stage (*p* = 0.044). The median OS was 30.75 months for stage I disease, 18.50 months for stage II disease, and 8.75 months for stage III and IV disease.

Surgical resection was significantly associated with OS (*p* = 0.001). Patients who underwent resection had a median OS of 31.25 months, compared with 8.25 months for those who did not undergo resection.

Adjuvant therapy was also significantly associated with OS (*p* < 0.001). The median OS was 35.25 months for patients who received adjuvant therapy and 7.75 months for those who did not (Supplementary Figure S1).

Univariate and multivariable Firth’s penalized Cox proportional hazards regression analyses were performed to evaluate factors associated with OS using the 10% CLDN18.2 positivity cutoff (Table 4). In the univariate analysis, absence of adjuvant therapy (hazard ratio (HR) = 3.361, 95% confidence interval (CI): 1.887–6.590, *p* < 0.001), absence of surgical resection (HR = 2.387, 95% CI: 1.426–4.274, *p* < 0.001), older age at diagnosis (HR = 1.020, 95% CI: 1.001–1.040, *p* = 0.042), and advanced AJCC stage (HR = 1.742, 95% CI: 1.115–2.830, *p* = 0.014) were significantly associated with increased mortality. CLDN18.2 positivity at the 10% cutoff was not significantly associated with mortality in the univariate analysis (HR = 1.641, 95% CI: 0.883–2.816, *p* = 0.112). In the multivariable model, absence of adjuvant therapy remained independently associated with increased mortality (HR = 4.870, 95% CI: 1.671–13.784, *p* = 0.004), as did older age at diagnosis (HR = 1.021, 95% CI: 1.002–1.042, *p* = 0.027). CLDN18.2 positivity at the 10% cutoff was also independently associated with an approximately two-fold increased risk of mortality (HR = 2.072, 95% CI: 1.090–3.666, *p* = 0.028). Neither surgical resection (*p* = 0.401) nor advanced AJCC stage (*p* = 0.552) remained independently associated with mortality in the multivariable model.

The CLDN18.2 positivity cutoff of 75% was also evaluated using univariate and multivariable Firth’s penalized Cox proportional hazards regression analyses (Table 5). In the univariate analysis, absence of adjuvant therapy (HR = 3.361, 95% CI: 1.887–6.590, *p* < 0.001), absence of surgical resection (HR = 2.387, 95% CI: 1.426–4.274, *p* < 0.001), older age at diagnosis (HR = 1.020, 95% CI: 1.001–1.040, *p* = 0.042), and advanced AJCC stage (HR = 1.742, 95% CI: 1.115–2.830, *p* = 0.014) were significantly associated with increased mortality. CLDN18.2 positivity at the 75% cutoff was not significantly associated with mortality in the univariate analysis (HR = 1.884, 95% CI: 0.762–3.877, *p* = 0.155). In the multivariable analysis, absence of adjuvant therapy (HR = 4.296, 95% CI: 1.521–11.899, *p* = 0.006) and older age at diagnosis (HR = 1.020, 95% CI: 1.001–1.040, *p* = 0.041) remained independently associated with increased mortality. In contrast to the 10% cutoff model, CLDN18.2 positivity at the 75% cutoff was not independently associated with mortality (HR = 1.906, 95% CI: 0.768–3.951, *p* = 0.150). Neither the absence of surgical resection (*p* = 0.404) nor advanced AJCC stage (*p* = 0.379) remained independently associated with mortality in the multivariable model.

## 4. Discussion

The demographic characteristics of our cohort, including a male predominance (59.7%) and a mean age at diagnosis of 61.0 years, are consistent with previous reports on CCA [1,2]. The predominance of iCCA in our series (87.9%) likely reflects the referral pattern to our institution, a tertiary hepatobiliary center. Although pCCA represents the most common anatomical subtype in population-based studies, accounting for approximately two-thirds of all CCA cases, surgical series from high-volume hepatobiliary centers consistently report a disproportionately higher proportion of iCCA because these tumors are more frequently amenable to hepatic resection [3].

Histologically, small-duct iCCA accounted for 74.2% of all cases, which is in agreement with previous surgical series, showing that this subtype comprises approximately 60–80% of resected iCCA cases [11].

The median OS of 9.5 months observed in our cohort is consistent with the advanced stage of disease in the study population, in which 42.7% of patients had AJCC stage IV disease. In the pivotal ABC-02 phase III trial, the combination of gemcitabine and cisplatin achieved a median OS of 11.7 months, highlighting the persistently poor prognosis of advanced CCA despite standard systemic therapy [4]. More recently, the updated analysis of the TOPAZ-1 trial demonstrated that the addition of durvalumab to gemcitabine–cisplatin improved median OS to 12.9 months [5]. However, these outcomes were derived from carefully selected clinical trial populations with good performance status, whereas shorter survival is expected in real-world cohorts, which are characterized by more advanced disease, greater clinical heterogeneity, and variable access to treatment.

Consistent with previous studies, Kaplan–Meier analysis demonstrated progressively shorter survival with advancing AJCC stage; median OS decreased from 30.75 months for stage I disease to 8.75 months for stage IV disease (*p* = 0.044). These findings are consistent with the well-established prognostic importance of tumor stage in CCA and underscore the clinical relevance of disease extent at diagnosis [12].

In our cohort, surgical resection status was significantly associated with OS. Patients who underwent curative-intent resection had a median OS of 31.25 months, compared with 8.25 months among those who did not undergo resection (*p* = 0.001). This association is consistent with previous reports. In the Memorial Sloan Kettering Cancer Center series, median OS was 37.4 months in patients who underwent resection compared with 11.6 months in those with unresectable disease (*p* = 0.006) [13]. Similarly, a multicenter study reported median OS values of 27.6 months for resected patients, 12.9 months for those receiving palliative chemotherapy, and 4.9 months for patients managed with best supportive care (*p* < 0.001) [14]. Nevertheless, the association between surgical resection and longer survival in retrospective cohorts should be interpreted in the context of treatment selection, as eligibility for resection is inherently influenced by disease stage, resectability, performance status, and other clinical factors.

In the multivariable Firth’s penalized Cox regression analyses, absence of adjuvant therapy was independently associated with increased mortality in both models (10% cutoff: HR = 4.870, 95% CI: 1.671–13.784, *p* = 0.004; 75% cutoff: HR = 4.296, 95% CI: 1.521–11.899, *p* = 0.006). This observation is consistent with previous randomized clinical trials and meta-analyses reporting an association between adjuvant therapy and improved outcomes following surgical resection for biliary tract cancer (BTC) [15,16]. However, the association observed in our cohort should not be interpreted as evidence of a causal effect of adjuvant therapy. Receipt of adjuvant therapy in a retrospective cohort may be influenced by multiple clinical factors, including disease stage, resectability, surgical treatment, postoperative recovery, performance status, and treatment eligibility. Notably, 84.7% of patients in our cohort did not receive adjuvant therapy. Therefore, the observed association between the absence of adjuvant therapy and poorer survival should be interpreted within the context of the retrospective, single-center study design and potential treatment-selection effects.

### 4.1. CLDN18.2 Expression Rates and the Methodological Importance of Positivity Thresholds

In the present study, CLDN18.2 expression was evaluated using two predefined positivity thresholds. At the 75% threshold, representing high-level expression, the positivity rate was 4.8% (6/124), whereas lowering the threshold to 10% increased the positivity rate to 10.5% (13/124). These findings are consistent with the range of positivity rates reported in previous studies employing comparable IHC criteria.

Using moderate-to-strong (2+/3+) membranous staining in ≥75% of tumor cells as the positivity criterion, Shin et al. reported a CLDN18.2 positivity rate of 5.2% in a large surgical cohort of 636 CCA cases, closely matching our findings [9]. In contrast, Hashimoto et al., using the multicenter MONSTAR-SCREEN-2 platform and a more permissive definition (any staining intensity in ≥40% of tumor cells), reported a positivity rate of 21.7% [17]. Similarly, Yu et al. evaluated CLDN18.2 expression in 83 surgically resected iCCA cases using an H-score ≥1, whereby membranous staining of any intensity in even a single tumor cell was considered positive, resulting in a positivity rate of 24.1% [18]. Employing an even broader definition, Yan et al. classified any membranous staining as positive and reported CLDN18.2 expression in 52% of CCA cases [19]. Likewise, in the screening program for CT041 anti-CLDN18.2 CAR-T cell therapy, XiaoYi et al. defined positivity as membranous staining of any intensity in ≥1% of tumor cells and reported a positivity rate of 65.6% among patients with biliary tract cancer [20].

Collectively, these studies demonstrate that the marked variability in reported CLDN18.2 positivity rates (approximately 5–66%) is likely attributable, at least in part, to differences in IHC scoring criteria, positivity thresholds, staining protocols, and study populations. Desai et al. further highlighted that the choice of positivity threshold directly influences both the reported prevalence of CLDN18.2 expression and its potential clinical applicability in CCA [10].

Accordingly, two positivity thresholds were evaluated in the present study. The 10% threshold was used to characterize a broader spectrum of CLDN18.2 expression that may be relevant to future CLDN18.2-targeted therapeutic strategies, whereas the 75% threshold corresponds to the high-expression definition adopted in contemporary therapeutic and regulatory studies of zolbetuximab. This dual-threshold approach provides a more comprehensive assessment of CLDN18.2 expression and facilitates comparison with the heterogeneous literature.

### 4.2. The Relationship Between Tumor Location and CLDN18.2 Expression

One notable finding of our study was the variation in CLDN18.2 expression across tumor anatomical locations. At the 75% positivity threshold, pCCA exhibited the highest positivity rate (30.0%), followed by iCCA (2.8%). No positive cases were identified in dCCA. CLDN18.2 positivity differed significantly by anatomical location (*p* = 0.013), and this distribution is consistent with previous reports.

Similarly, Shin et al. reported CLDN18.2 positivity rates of 22.2% for pCCA, 1.6% for iCCA, and 5.6% for dCCA in a large cohort of 636 patients, demonstrating a significantly higher prevalence in pCCA than in the other anatomical subtypes [9].

The biological basis for this distribution remains to be fully elucidated. However, this may reflect differences in molecular characteristics and epithelial differentiation programs across the anatomical subtypes of CCA. Compared with iCCA and dCCA, pCCA may retain a distinct epithelial phenotype favoring CLDN18.2 expression, although the mechanisms underlying this association require further investigation.

From a clinical perspective, the higher prevalence of CLDN18.2 expression observed in pCCA in our cohort, together with the findings reported by Shin et al. [9], suggests that this subgroup may represent a relevant population for further investigation of CLDN18.2-targeted therapeutic strategies and prospective clinical trials. However, given the relatively small number of pCCA cases in our cohort, this observation should be considered preliminary and interpreted with appropriate caution. Confirmation in larger, independent, and preferably multicenter cohorts is warranted.

### 4.3. The Relationship Between Histological Subtype and CLDN18.2 Expression

In our study, CLDN18.2 expression differed significantly by histological subtype (*p* = 0.002). Using a 75% positivity threshold, 21.7% of large-duct iCCA cases were CLDN18.2-positive, compared with 1.1% of small-duct iCCA cases, and no positive cases were identified among cHCC–CCA cases. Although these findings suggest a potential association between histological subtype and CLDN18.2 expression, the relatively small number of patients in the less frequent subtypes warrants cautious interpretation.

This distribution is biologically plausible given the distinct histogenetic and molecular characteristics of these tumor subtypes. Large-duct iCCA arises from the epithelium of the large intrahepatic bile ducts and is characterized by mucin-producing columnar cells and biliary-type glandular differentiation [1,11]. These histopathological features resemble those of other gastrointestinal adenocarcinomas for which CLDN18.2 expression is well established, such as gastric and pancreatic ductal adenocarcinomas.

In contrast, small-duct iCCA is believed to originate from the peripheral bile ducts or hepatic progenitor cells and is characterized by a distinct molecular landscape enriched for IDH1/2, BAP1, and FGFR2 alterations [3,11,21]. These fundamental biological differences may contribute to the lower frequency of CLDN18.2 expression observed in this subtype.

No CLDN18.2-positive cases were identified among cHCC–CCA tumors. Although the number of cases was limited, this finding may be related to the mixed hepatocellular and cholangiocytic differentiation that characterizes these neoplasms and may contribute to their distinct immunophenotypic profile [1].

Overall, observed differences in CLDN18.2 expression across histological subtypes suggest that histological classification may be relevant to selecting patients for future CLDN18.2-targeted therapeutic strategies and clinical trials. However, the relatively small numbers of patients in the less frequent subtypes, particularly large-duct iCCA and cHCC–CCA, warrant cautious interpretation and should be considered preliminary. Further validation in larger, independent, and preferably multicenter cohorts is needed to determine whether the observed differences are reproducible and to establish more reliable estimates of prevalence across histological subtypes.

### 4.4. Association with Other Clinicopathological Parameters

In our study, CLDN18.2 expression was not significantly associated with established clinicopathological prognostic factors, including histological grade, LVI, PNI multifocality, AJCC stage, T stage, N stage, M stage, portal vein invasion, or the number of metastatic foci.

Nevertheless, several numerical differences were observed. CLDN18.2 positivity was more frequent in tumors without LVI than in those with LVI, although this difference did not reach statistical significance (*p* = 0.088). Likewise, unifocal tumors showed a higher positivity rate than multifocal tumors (*p* = 0.085). Given the lack of statistical significance, these observations should be considered exploratory and do not permit definitive conclusions about the relationship between CLDN18.2 expression and tumor invasiveness.

Yu et al. reported that CLDN18.2-positive iCCAs were associated with smaller tumor size and unifocal disease, suggesting a potentially distinct pattern of tumor progression and metastatic behavior [18]. Consistent with these observations, the mean tumor size in our cohort was numerically smaller in CLDN18.2-positive patients (55.0 mm) than in CLDN18.2-negative patients (75.7 mm), although this difference was not statistically significant (*p* = 0.208).

Taken together, these observations raise the possibility that CLDN18.2 expression may be associated with specific biological characteristics that are not fully captured by conventional clinicopathological variables. However, given the lack of statistical significance in our cohort, these potential associations remain exploratory and require confirmation in larger, independent studies.

### 4.5. Prognostic Value of CLDN18.2

In the univariate Firth’s penalized Cox regression analyses, CLDN18.2 positivity was not significantly associated with mortality at either the 10% cutoff (HR = 1.641, 95% CI: 0.883–2.816; *p* = 0.112) or the 75% cutoff (HR = 1.884, 95% CI: 0.762–3.877; *p* = 0.155). However, a different pattern emerged in the multivariable analyses. At the 10% cutoff, CLDN18.2 positivity was independently associated with an approximately twofold higher risk of mortality (HR = 2.072, 95% CI: 1.090–3.666; *p* = 0.028). In contrast, CLDN18.2 positivity at the 75% cutoff was not independently associated with mortality (HR = 1.906, 95% CI: 0.768–3.951; *p* = 0.150).

These findings have two potentially important implications. First, differences between the results obtained using the 10% and 75% positivity thresholds suggest that the selected threshold may influence the prognostic assessment of CLDN18.2 expression. This observation underscores the importance of establishing standardized scoring criteria and clinically relevant positivity thresholds for CLDN18.2 evaluation in CCA. Second, the association between CLDN18.2 positivity at the 10% cutoff and increased mortality after multivariable adjustment, despite the lack of a significant association in the univariate analysis, suggests that its prognostic value may become more apparent after accounting for other clinicopathological factors. Nevertheless, this finding should be interpreted cautiously and requires validation in independent cohorts.

Published data on the prognostic significance of CLDN18.2 in CCA remain inconsistent. In a surgical series of 83 iCCA cases, Yu et al. identified CLDN18.2 positivity as an independent predictor of OS (HR = 2.555, 95% CI: 1.250–5.223; *p* = 0.01) [18]. Similarly, Kuo et al. reported that CLDN18.2 overexpression in 182 iCCA patients was associated with R1 resection (*p* = 0.032) and advanced T stage (*p* = 0.043), and demonstrated an independent adverse prognostic association in multivariable analysis [22]. In contrast, Shin et al. found no significant association between CLDN18.2 expression and either OS or disease-free survival in a large cohort of 636 CCA cases [9]. These discrepancies may reflect differences in patient populations, anatomical subtype distribution, IHC scoring systems, positivity thresholds, treatment strategies, and duration of follow-up. Our findings add to this heterogeneous literature by suggesting that the prognostic association of CLDN18.2 may vary according to the positivity threshold applied, with an independent association with mortality observed at the 10% cutoff but not at the 75% cutoff.

### 4.6. Therapeutic Implications of CLDN18.2 Expression in Cholangiocarcinoma

The strongest clinical evidence supporting CLDN18.2-targeted therapy currently comes from patients with advanced gastric and gastroesophageal junction adenocarcinomas. In the phase III SPOTLIGHT trial, the addition of zolbetuximab to mFOLFOX6 significantly improved both PFS and OS in patients with CLDN18.2-positive/HER2-negative tumors compared with placebo plus chemotherapy [7]. These findings were further supported by the phase III GLOW trial, in which zolbetuximab combined with CAPOX significantly prolonged PFS and OS compared with placebo plus CAPOX [23]. Collectively, these landmark studies have established CLDN18.2 as a clinically actionable therapeutic target in gastric and gastroesophageal junction adenocarcinomas and have provided a rationale for investigating CLDN18.2-directed strategies in other CLDN18.2-expressing malignancies.

In CCA, however, the available evidence remains largely limited to expression profiling and biomarker characterization. Nevertheless, accumulating data provide a biological rationale for further evaluation of CLDN18.2-directed therapies in this disease. Hashimoto et al. identified CLDN18.2 expression in CCA within the MONSTAR-SCREEN-2 platform, supporting further investigation of this biomarker in CCA [17]. Likewise, Shin et al. proposed CLDN18.2, together with Trop-2, as a potential target for antibody–drug conjugate (ADC)-based therapies and emphasized the need for dedicated clinical studies in biliary tract cancers [9]. Furthermore, the CT041 anti-CLDN18.2 CAR-T screening program reported CLDN18.2 positivity in 65.6% of patients with biliary tract cancer using a highly permissive biomarker definition, illustrating how the proportion of patients classified as CLDN18.2-positive may vary substantially according to the positivity criteria applied [20].

In the present study, the relatively high CLDN18.2 positivity rates observed in pCCA (30.0%) and large-duct iCCA (21.7%) suggest that these subgroups may represent relevant populations for further investigation in biomarker-driven clinical studies. However, given the limited number of cases in these subgroups, these observations should be considered hypothesis-generating rather than definitive evidence for patient selection. Prospective multicenter studies and interventional clinical trials will be essential to determine whether CLDN18.2 expression can serve as a clinically meaningful therapeutic biomarker in CCA.

### 4.7. Limitations of the Study

This study has several limitations that should be considered when interpreting the findings. First, its retrospective, single-center design may have introduced selection and information biases. In addition, the predominance of iCCA (87.9%) resulted in relatively small pCCA and dCCA subgroups, thereby limiting the statistical power and generalizability of subgroup-specific analyses. Similarly, the relatively small numbers of large-duct iCCA and cHCC–CCA cases limit the precision of subtype-specific estimates. Consequently, the relatively high CLDN18.2 positivity rates observed in pCCA (30.0%) and large-duct iCCA (21.7%) should be interpreted with caution and regarded as preliminary findings that require validation in larger, independent cohorts.

Another important limitation is that CLDN18.2 expression was evaluated exclusively by immunohistochemistry, without complementary molecular validation using techniques such as fluorescence in situ hybridization (FISH), next-generation sequencing (NGS), or RNA expression profiling. In addition, the absence of standardized antibody clones, staining protocols, and scoring systems across published studies complicates direct comparisons between cohorts.

Finally, the high mortality rate (88.7%), short median OS (9.5 months), and heterogeneity in treatment approaches may have influenced the survival analyses and should be considered when interpreting the prognostic findings. In particular, the observed association between the absence of adjuvant therapy and poorer survival should be interpreted as an observational association rather than evidence of a causal treatment effect. Receipt of adjuvant therapy may have been influenced by multiple clinical factors, including disease stage, resectability, surgical treatment, postoperative recovery, performance status, and treatment eligibility, resulting in potential treatment-selection bias and residual confounding. Therefore, prospective multicenter studies, including larger and more balanced cohorts across CCA subtypes and using standardized methods for CLDN18.2 assessment, are warranted to validate and extend the present findings.

## 5. Conclusions

This study provides a comprehensive real-world assessment of CLDN18.2 expression in CCA and demonstrates that its expression varies by anatomical location, histological subtype, and the positivity threshold applied. The relatively high expression rates observed in pCCA and large-duct iCCA, together with the independent association between CLDN18.2 positivity at the 10% threshold and increased mortality identified by Firth’s penalized Cox regression, suggest a potential role for CLDN18.2 as a prognostic biomarker and therapeutic target for selected patients with CCA. Given the relatively small number of patients in some anatomical and histological subgroups, the observed differences in CLDN18.2 expression should be considered preliminary and interpreted with caution. These findings provide a rationale for further evaluation of CLDN18.2 in biomarker-driven clinical trials and for validation in large, prospective, multicenter studies that employ standardized assessment methods.

## Figures and Tables

**Table 1:** Baseline clinicopathological characteristics of the study cohort.

		*n*	%
Histological subtype	Small-duct iCCA	92	74.2
	Large-duct iCCA	23	18.5
	cHCC–CCA	9	7.3
Histological Grade	Grade 1	36	29.0
	Grade 2	66	53.2
	Grade 3	22	17.7
Lymphovascular invasion	Absent	11	8.9
	Present	113	91.1
Perineural invasion	Absent	23	18.5
	Present	101	81.5
Multifocality	Absent	40	32.3
	Present	84	67.7
AJCC stage (8th edition)	1A	4	3.2
	1B	3	2.4
	2	24	19.4
	3	40	32.3
	4	53	42.7
T stage	1A	8	6.5
	1B	4	3.2
	2	92	74.2
	3	6	4.8
	4	14	11.3
N stage	0	34	27.4
	1	88	71.0
	2	2	1.6
M stage	0	71	57.3
	1	53	42.7
Metastatic disease	Unifocal	28	52.8
	Multifocal	25	47.2
Portal Vein Invasion	Absent	103	83.1
	Present	21	16.9
Neoadjuvant Therapy	Absent	111	89.5
	Present	13	10.5
Surgical Resection	Absent	101	81.5
	Present	23	18.5
CLDN18.2 expression (75% Cutoff)	Negative	118	95.2
	Positive	6	4.8
CLDN18.2 expression (10% Cutoff)	Negative	111	89.5
	Positive	13	10.5
Locoregional Therapy	None	80	64.5
	TARE	43	34.7
	TACE	1	0.8
Adjuvant Therapy	Absent	105	84.7
	Present	19	15.3
Overall Survival (OS)	Alive	14	11.3
	Dead	110	88.7

Abbreviations: iCCA, intrahepatic cholangiocarcinoma; cHCC–CCA, hepatocellular–cholangiocarcinoma; AJCC, American Joint Committee on Cancer; TARE, transarterial radioembolization; TACE, transarterial chemoembolization; CLDN18.2, Claudin 18.2.

**Table 2:** Association between CLDN18.2 expression (75% positivity threshold) and clinicopathological characteristics.

		CLDN18.2 Expression (75% Cutoff)		*p*
		Negative	Positive	
		*n* (%)	*n* (%)	
Gender	Male	69 (93.2)	5 (6.8)	0.400
	Female	49 (98)	1 (2)	
Anatomical location	iCCA	106 (97.2)	3 (2.8)	**0.013**
	pCCA	7 (70)	3 (30)	
	dCCA	5 (100)	0 (0)	
Histological subtype	Small-duct iCCA	91 (98.9)	1 (1.1)	**0.002**
	Large-duct iCCA	18 (78.3)	5 (21.7)	
	cHCC–CCA	9 (100)	0 (0)	
Histological Grade	Grade 1	34 (94.4)	2 (5.6)	0.730
	Grade 2	62 (93.9)	4 (6.1)	
	Grade 3	22 (100)	0 (0)	
Lymphovascular invasion	Absent	9 (81.8)	2 (18.2)	0.088
	Present	109 (96.5)	4 (3.5)	
Perineural invasion	Absent	21 (91.3)	2 (8.7)	0.308
	Present	97 (96)	4 (4)	
Multifocality	Absent	36 (90)	4 (10)	0.085
	Present	82 (97.6)	2 (2.4)	
AJCC stage (8th edition)	1A	3 (75)	1 (25)	0.378
	1B	3 (100)	0 (0)	
	2	23 (95.8)	1 (4.2)	
	3	39 (97.5)	1 (2.5)	
	4	50 (94.3)	3 (5.7)	
T stage	1A	7 (87.5)	1 (12.5)	0.686
	1B	4 (100)	0 (0)	
	2	87 (94.6)	5 (5.4)	
	3	6 (100)	0 (0)	
	4	14 (100)	0 (0)	
N stage	0	32 (94.1)	2 (5.9)	0.702
	1	84 (95.5)	4 (4.5)	
	2	2 (100)	0 (0)	
M stage	0	68 (95.8)	3 (4.2)	0.713
	1	50 (94.3)	3 (5.7)	
Metastatic disease	Unifocal	27 (96.4)	1 (3.6)	0.597
	Multifocal	23 (92)	2 (8)	
Portal Vein Invasion	Absent	97 (94.2)	6 (5.8)	0.588
	Present	21 (100)	0 (0)	
Neoadjuvant Therapy	Absent	106 (95.5)	5 (4.5)	0.493
	Present	12 (92.3)	1 (7.7)	
Surgical Resection	Absent	96 (95)	5 (5)	0.903
	Present	22 (95.7)	1 (4.3)	
Locoregional Therapy	None	76 (95)	4 (5)	0.971
	TARE	41 (95.3)	2 (4.7)	
	TACE	1 (100)	0 (0)	
Adjuvant Therapy	Absent	100 (95.2)	5 (4.8)	0.925
	Present	18 (94.7)	1 (5.3)	

Abbreviations: iCCA, intrahepatic cholangiocarcinoma; pCCA, perihilar cholangiocarcinoma; dCCA, distal cholangiocarcinoma; cHCC–CCA, hepatocellular–cholangiocarcinoma; AJCC, American Joint Committee on Cancer; TARE, transarterial radioembolization; TACE, transarterial chemoembolization; CLDN18.2, Claudin 18.2. Bold values indicate statistical significance (*p* < 0.05).

**Table 3:** Association of clinicopathological features and treatment parameters with patient survival status.

		Mortality		*p*
		Alive	Dead	
		*n* (%)	*n* (%)	
Gender	Male	8 (10.8)	66 (89.2)	0.837
	Female	6 (12)	44 (88)	
Anatomical location	iCCA	12 (11)	97 (89)	0.638
	pCCA	1 (10)	9 (90)	
	dCCA	1 (20)	4 (80)	
Histological subtype	Small-duct iCCA	9 (9.8)	83 (90.2)	0.192
	Large-duct iCCA	5 (21.7)	18 (78.3)	
	cHCC–CCA	0 (0)	9 (100)	
Histological Grade	Grade 1	7 (19.4)	29 (80.6)	0.214
	Grade 2	6 (9.1)	60 (90.9)	
	Grade 3	1 (4.5)	21 (95.5)	
Lymphovascular invasion	Absent	3 (27.3)	8 (72.7)	0.110
	Present	11 (9.7)	102 (90.3)	
Perineural invasion	Absent	5 (21.7)	18 (78.3)	0.135
	Present	9 (8.9)	92 (91.1)	
Multifocality	Absent	6 (15)	34 (85)	0.377
	Present	8 (9.5)	76 (90.5)	
AJCC stage (8th edition)	1A	2 (50)	2 (50)	**<0.001**
	1B	2 (66.7)	1 (33.3)	
	2	4 (16.7)	20 (83.3)	
	3	5 (12.5)	35 (87.5)	
	4	1 (1.9)	52 (98.1)	
T stage	1A	3 (37.5)	5 (62.5)	**0.017**
	1B	2 (50)	2 (50)	
	2	9 (9.8)	83 (90.2)	
	3	0 (0)	6 (100)	
	4	0 (0)	14 (100)	
N stage	0	8 (23.5)	26 (76.5)	**0.004**
	1	5 (5.7)	83 (94.3)	
	2	1 (50)	1 (50)	
M stage	0	13 (18.3)	58 (81.7)	**0.004**
	1	1 (1.9)	52 (98.1)	
Metastatic disease	Unifocal	1 (3.6)	27 (96.4)	0.340
	Multifocal	0 (0)	25 (100)	
Portal Vein Invasion	Absent	14 (13.6)	89 (86.4)	0.073
	Present	0 (0)	21 (100)	
Neoadjuvant therapy	Absent	13 (11.7)	98 (88.3)	0.665
	Present	1 (7.7)	12 (92.3)	
Surgical resection	Absent	6 (5.9)	95 (94.1)	**<0.001**
	Present	8 (34.8)	15 (65.2)	
CLDN18.2 expression (75% Cutoff)	Negative	14 (11.9)	104 (88.1)	0.370
	Positive	0 (0)	6 (100)	
CLDN18.2 expression (10% Cutoff)	Negative	14 (12.6)	97 (87.4)	0.358
	Positive	0 (0)	13 (100)	
Locoregional Therapy	None	9 (11.3)	71 (88.8)	0.936
	TARE	5 (11.6)	38 (88.4)	
	TACE	0 (0)	1 (100)	
Adjuvant therapy	Absent	6 (5.7)	99 (94.3)	**<0.001**
	Present	8 (42.1)	11 (57.9)	

Abbreviations: iCCA, intrahepatic cholangiocarcinoma; pCCA, perihilar cholangiocarcinoma; dCCA, distal cholangiocarcinoma; cHCC–CCA, hepatocellular–cholangiocarcinoma; AJCC, American Joint Committee on Cancer; TARE, transarterial radioembolization; TACE, transarterial chemoembolization; CLDN18.2, Claudin 18.2. Bold values indicate statistical significance (*p* < 0.05).

**Table 4:** Univariate and multivariable Firth’s penalized Cox proportional hazards regression analysis of OS according to CLDN18.2 10% positivity thresholds.

	Univariate	*p*	Multivariate	*p*
	HR (95% CI)		HR (95% CI)	
No adjuvant therapy	3.361 (1.887–6.590)	**<0.001**	4.870 (1.671–13.784)	**0.004**
No surgical resection	2.387 (1.426–4.274)	**<0.001**	0.675 (0.303–1.784)	0.401
Age at diagnosis	1.020 (1.001–1.040)	**0.042**	1.021 (1.002–1.042)	**0.027**
Advanced AJCC stage	1.742 (1.115–2.830)	**0.014**	1.157 (0.724–1.918)	0.552
CLDN18.2 positivity	1.641 (0.883–2.816)	0.112	2.072 (1.090–3.666)	**0.028**

HR, hazard ratio; CI, confidence interval; AJCC, American Joint Committee on Cancer; CLDN18.2, Claudin 18.2. Bold values indicate statistical significance (*p* < 0.05).

**Table 5:** Univariate and multivariable Firth’s penalized Cox proportional hazards regression analysis of OS according to CLDN18.2 75% positivity thresholds.

	Univariate	*p*	Multivariate	*p*
	HR (95% CI)		HR (95% CI)	
No adjuvant therapy	3.361 (1.887–6.590)	**<0.001**	4.296 (1.521–11.899)	**0.006**
No surgical resection	2.387 (1.426–4.274)	**<0.001**	0.681 (0.309–1.761)	0.404
Age at diagnosis	1.020 (1.001–1.040)	**0.042**	1.020 (1.001–1.040)	**0.041**
Advanced AJCC stage	1.742 (1.115–2.830)	**0.014**	1.237 (0.777–2.045)	0.379
CLDN18.2 positivity	1.884 (0.762–3.877)	0.155	1.906 (0.768–3.951)	0.150

HR, hazard ratio; CI, confidence interval; AJCC, American Joint Committee on Cancer; CLDN18.2, Claudin 18.2. Bold values indicate statistical significance (*p* < 0.05).

## Data Availability

Data supporting the findings of this study are available from the corresponding author upon rea-sonable request. The data were not publicly available because of ethical and privacy restrictions protecting patient confidentiality.

## Supplementary Materials

The following supporting information can be downloaded at: https://www.mdpi.com/article/10.3390/jcm15176621/s1, Figure S1: Kaplan–Meier curves for overall survival according to (A) AJCC stage, (B) surgical resection status, and (C) adjuvant therapy status.

## Abbreviations

The following abbreviations are used in this manuscript:

CLDN18.2	Claudin 18.2
CCA	Cholangiocarcinoma
iCCA	Intrahepatic cholangiocarcinoma
pCCA	Perihilar cholangiocarcinoma
dCCA	Distal cholangiocarcinoma
cHCC–CCA	Hepatocellular–cholangiocarcinoma
OS	Overall survival
PFS	Progression-free survival
IHC	Immunohistochemical
AJCC	The American Joint Committee on Cancer
