# Supplementary material for: Claudin 18.2 Expression in Cholangiocarcinoma: Clinicopathological Heterogeneity, Prognostic Significance, and Impact of Positivity Thresholds
Source: J Clin Med. 2026 Aug 27;15(17):6621. doi: 10.3390/jcm15176621 (PMC13566907; doi:10.3390/jcm15176621)
Supplement: Supplementary file 1 [file jcm-15-06621-s001.zip › jcm-4466592-supplementary.pdf]

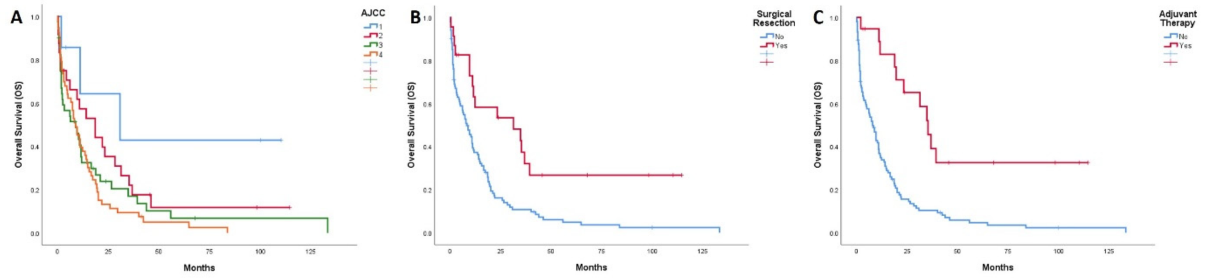

Figure S1. Kaplan–Meier curves for overall survival according to (A) AJCC stage, (B) surgical resection status, and (C) adjuvant therapy status.
